# Saccharated Calomel

**Published:** 1874-05

**Authors:** 


### SACCHARATED CALOMEL.

*Editor Atlanta Medical Journal:*

In regard to "Saccharated Calomel," I write to say that in 1858, my venerable friend, Dr. Samuel K. Jennings, of Selma, Alabama, drew my attention to the half-grain doses of calomel, stating its remarkable cholagogue effects in so small a quantity. Given at night, it always produced from one to four billious actions from the bowels. This was his

℞—Calomel, . . . . . . . gr. j.
Loaf Sugar, . . . . . . . gr. ij.
Mix and make two pills.

Dose one every other night. During an active practice I used minute doses of calomel, and during the four years of army practice in the field, I continued these small doses without the sugar, and with the happiest effect. When a soldier came to me I gave him a half-grain dose, placed far back on his tongue, and instructed him to swallow all the saliva secreted at the time. Without giving the rationale, I believe this the best mode of giving calomel in civil or army practice. I believe my friend, Dr.

Carlisle Terry, of Columbus, Georgia, still gives his calomel in this manner, though I think he gives two to three grains. I honestly believe a half-grain sufficient. Doctor Sam. K. Jennings was in 1858, seventy-two years old, and said to me he had used these small doses for forty years at that date. He has a written paper on this subject, that I will try to send you. Dr. Jennings was a true Allopath, no Hahnemanian. I mention the above facts in order that you may use them as you see fit. B.

LOUISVILLE, KY., March, 1874.

